# Morphological study of mechanoreceptors in collateral ligaments of the ankle joint

**DOI:** 10.1186/s13018-015-0215-7

**Published:** 2015-06-12

**Authors:** Xiaochuan Wu, Weidong Song, Cuihuan Zheng, Shixiong Zhou, Shengbin Bai

**Affiliations:** Department of Orthopaedics, Sanxiang Hospital, Zhongshan, Guangdong Province 528463 China; Department of Orthopaedics, Sun Yat-sen Memorial Hospital, 107 Riverside Road, Guangzhou, Guangdong Province 510120 China; Intensive Care Unit, Sanxiang Hospital, Zhongshan, Guangdong Province 528463 China; Department of Histology and Embryology, Guangdong Medical College, Zhanjiang, Guangdong Province 523000 China; Department of Histology and Embryology, Xinjiang Medical University, Urumqi, Xinjiang Province 830054 China

**Keywords:** Morphology, Mechanoreceptors, Collateral ligaments, Ankle joint, Pacinian corpuscles

## Abstract

**Background:**

The aim of this study was to analyze the pattern and types of sensory nerve endings in ankle collateral ligaments using histological techniques, in order to observe the morphology and distribution of mechanoreceptors in the collateral ligaments of cadaver ankle joint, and to provide the morphological evidence for the role of the ligament in joint sensory function.

**Methods:**

Twelve lateral collateral ligaments including anterior talofibular ligament (ATFL; *n* = 6), posterior talofibular ligament (PTFL; *n* = 6), and calcaneofibular ligament (CFL; *n* = 6) were harvested from six fresh frozen cadavers. The ligaments were embedded in paraffin, sectioned at 4 μm, and then stained using a modified gold-chloride staining methods. The collateral ligament was divided into three segments: proximal, middle, and distal segments. Fifty-four ATFL slides, 90 PTFL slides, and 108 CFL slides were analyzed. Mechanoreceptors were classified based on Freemen and Wyke’s classification. Mechanoreceptor distribution was analyzed statistically. One-way ANOVA (postHoc LSD) was used for statistical analysis.

**Results:**

All the four typical types of nerve endings (the Ruffini corpuscles, Pacinian corpuscles, Golgi tendon organs, and free nerve endings) were identified in these ligaments. Pacinian corpuscles were the predominant in all four complexes. More mechanoreceptors were found in synovial membrane near both ends of the ligaments attached to the bone. No statistical differences were found in the amount of mechanoreceptors among distal, middle, and proximal parts of the ligaments.

**Conclusions:**

The four typical types of mechanoreceptors were all identified in the collateral ligaments of the human ankle. Pacinian corpuscles were the predominant in all four complexes. This indicates that the main function of ankle collateral ligaments is to sense joint speeds in motions.

## Introduction

Ankles are the most important large joints of shock absorption and load bearing. The ankle sprain is one of the most common sports injuries in clinic [1]. It happens when stepping on uneven ground or on some object, with the movement of excessive plantar flexion and ankle inversion. As the lateral ligaments are weaker than the medial ligament and the inversion muscle group is stronger than the eversion muscle group, the lateral ligaments are likely to be injured, taking up about 85 % of the ankle sprain [2]. As the collateral ligaments play an important role in the structural stability of the ankle, the biomechanical stability will be disturbed after injury.

In addition to biomechanical function, the collateral ligaments also contribute to the proprioceptive function [3, 4]. Once injured, it can disturb not only the proprioceptive information input but also the coordination control of the nerve center to the complex of the joint nerve muscle, leading to functional instability of the ankle [5]. In clinic, the main purpose to treat ankle sprain and ankle joint instability is to restore the structural stability of the ankle. There are dozens of operation methods, while the outcome is not satisfied [6]. It has been reported that the curative satisfactory outcomes of lateral ligament injury are correlated to functional instability, not the mechanical instability [7]. As the importance of restoring the proprioceptive function has long been neglected, the chance of chronic ankle instability increases greatly [8, 9]. To better understand the function of the ankle collateral ligament, the microcosmic studies of the mediating organs in the ligament have been performed [10–14]. The proprioceptive loss and restore in lateral ankle sprain (LAS) and chronic ankle instability (CAI) are becoming a hot spot in the sports medicine and orthopedics research [15, 16]. The morphology, classification, distribution, and mechanisms have not completely reached consensus, which hinders the treatment research of the functional instability. The purpose of this study was to understand neural anatomic function of the ankle collateral ligament, so that to provide rationale for joint surgical treatment.

This study focused on the morphological structure and distribution of the proprioceptive mechanoreceptors in the lateral ligaments of the human ankle. Gold-chloride staining was used after paraffin sectioning. The purpose of this study is to provide some morphological evidence for the clinical treatment of the LAS and CAI.

## Material and methods

The collateral ligaments of ankle joints were harvested from six fresh frozen cadavers, including four males and two females, with the age of 21 to 45 years old. No arthrotrauma, degeneration, and nervous system diseases were found. The ligaments were harvested 12 h after cadavers were thawed at room temperature. This study was approved by the ethics committee of Department of Orthopaedics, Sun Yat-sen Memorial Hospital.

The lateral malleolus was exposed to identify end points of the ligaments attached to the bones (fibula and calcaneus). The ligaments were cut closely to the bone attached region at both ends with synovial tissues of the ligaments reserved. The ligaments were rinsed in sterile distilled water for less than 30 min and then cut into distal, middle, and proximal segments. The harvested ligament was divided into three segments equally, with segment attached to the fibula was defined as proximal segment. Different parts were marked by threads with different lengths. The orientations of ligaments were also marked. The ligaments were embedded using paraffin. Four μm thick of cross sections of the ligaments were made. After sections were dehydrated in alcohol, the modified O’Connor and Gonzales gold-chloride staining method [5] was used to stain the sectioned ligament tissues.

Same number of sections was selected from the same ligament according to the distance interval. As a result, 54 anterior talofibular ligament (ATFL), 90 posterior talofibular ligament (PTFL), and 108 calcaneofibular ligament (CFL) sections were obtained, including 84 sections of each of the distal, middle, and proximal parts. The morphologies of mechanoreceptors were observed under light microscope. The classification of sensory receptors was based on the classification proposed by Freeman and Wyke [17], which classified mechanoreceptors into four types according to the morphology and function (Table 1). The mechanoreceptors of each type in every selected section were counted. Then one-way ANOVA (postHoc, LSD) was performed using SPSS software (Ver. 15) to compare the mean value of each type of sensory receptor in different segments of a ligament. *P* < 0.05 was considered to be statistically significant.

**Table 1:** Classification of mechanoreceptors, by Freeman and Wyke [17]

Type	Name	Feature	Size (μm)	Characteristics
I	Ruffini	Round shaped, thinly myelinated, connected by nerve fibers;	100 × 40	Low threshold, slowly adapted
II	Pacini	Column or cone shaped, thickly myelinated;	280 × 120	Low threshold, rapidly adapted
III	Golgi	Spindle shaped, thinly myelinated, connected by thicker nerve fibers	600 × 100	Low threshold, slowly adapted
IV	Free nerve endings	Non-myelinated, irregular;	diameter 0.5 ~ 5	Transmit nociceptive sensation

## Result

### Overall outcomes of gold-chloride staining

#### Morphology observation

**Fig. 1:**
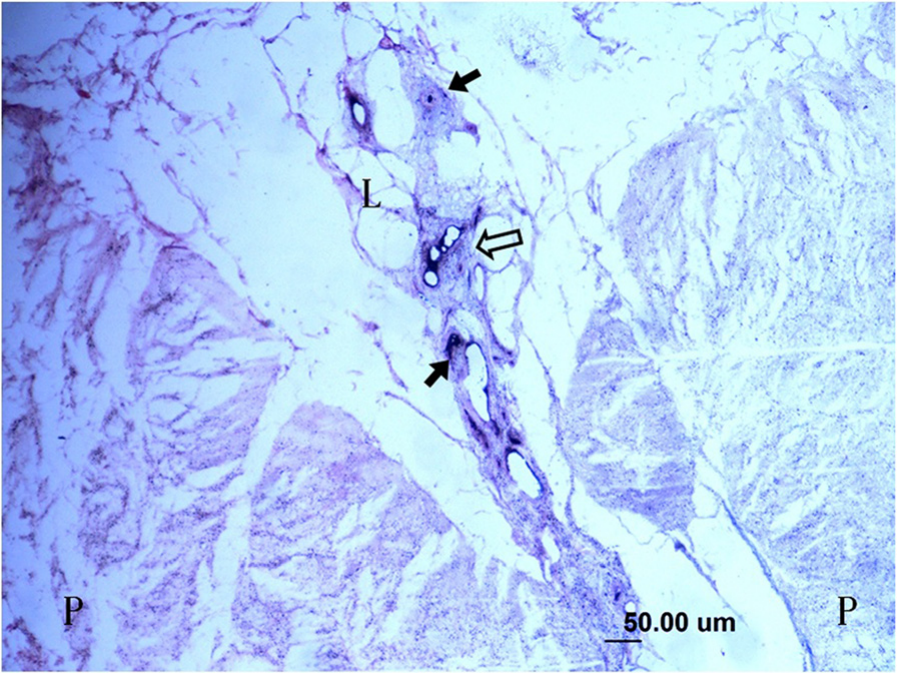
Sensory receptors (*arrow*), blood vessels (*hollow arrow*), adipose and loose connective tissues (*L*) in the parenchymal (*P*) tissues of ligament and synovial tissue (×100)

**Fig. 2:**
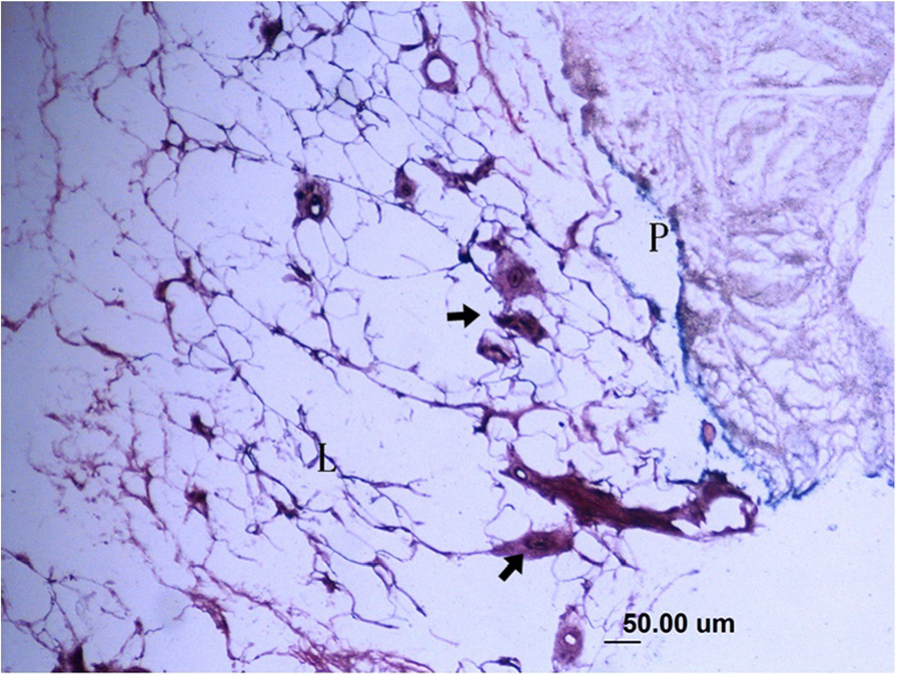
Several sensory receptors (*arrow*) were found in loose connective tissue (*L*) that attached to the bone, they mainly are Pacini corpuscles (×100)

In this study, the collagenous fibers were stained in light purple or light red, and the fibroblasts were also dyed in a stronger purple or red color with vague boundaries. The nerve fiber and the axons that connected the mechanoreceptor corpuscles were stained in darker red color close to black color. The synovial tissues, which consisted of loose connective tissues, were dyed lightly. In the synovial tissues, the arteries, veins, nerve fibers, and receptor corpuscles were found (Figs. 1 and 2). Abundance of vacuoles adipose tissue was found in the synovial tissues and ligaments.

#### Morphology and classification of mechanoreceptors

According to the classification of Freeman and Wyke, the four types of mechanoreceptors were identified in the collateral ligaments of the ankle, which were Ruffini (type I), Pacini (type II), Golgi (type III), and free nerve endings (type IV).

**Fig. 3:**
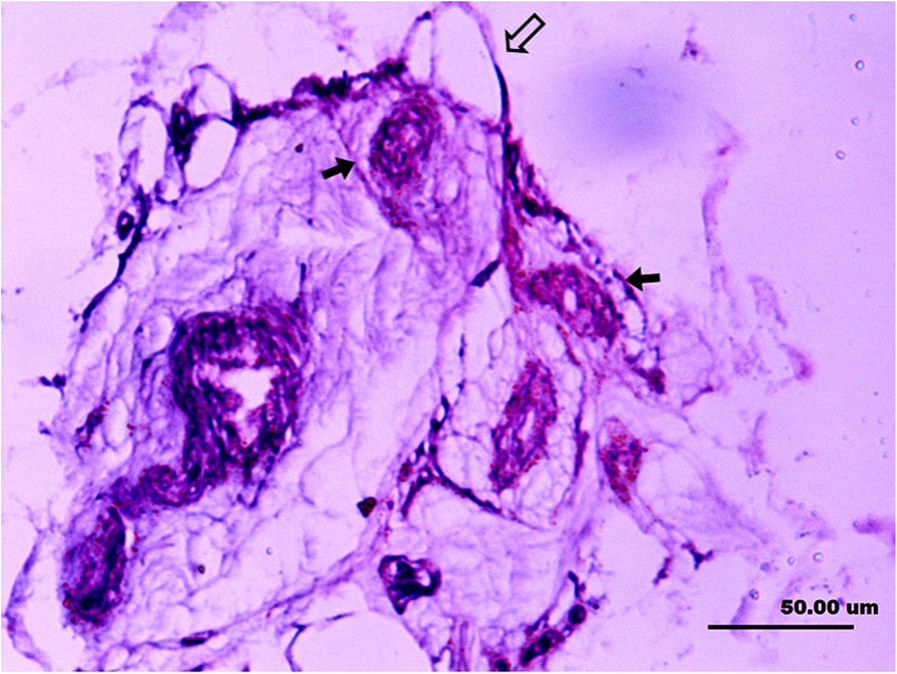
Ruffini (type I) and associated dendritic nerve fibers (*hollow arrow*) and spindle-shaped corpuscle (*solid arrow*) (×400)

The Ruffini (Fig. 3) formed dendritic nerve endings and ended at thinly myelinated spindle corpuscle. They were dyed bluish violet, forming a cluster of two to five corpuscles with 60 × 25 μm, which were found in all ligament samples. The axons attached to the corpuscles were dyed lighter black color, with a diameter of 3–5 μm. The capillaries were found within nerve fibers giving rise to Ruffini, which was different from a classical type I mechanoreceptors (Fig. 3).

**Fig. 4:**
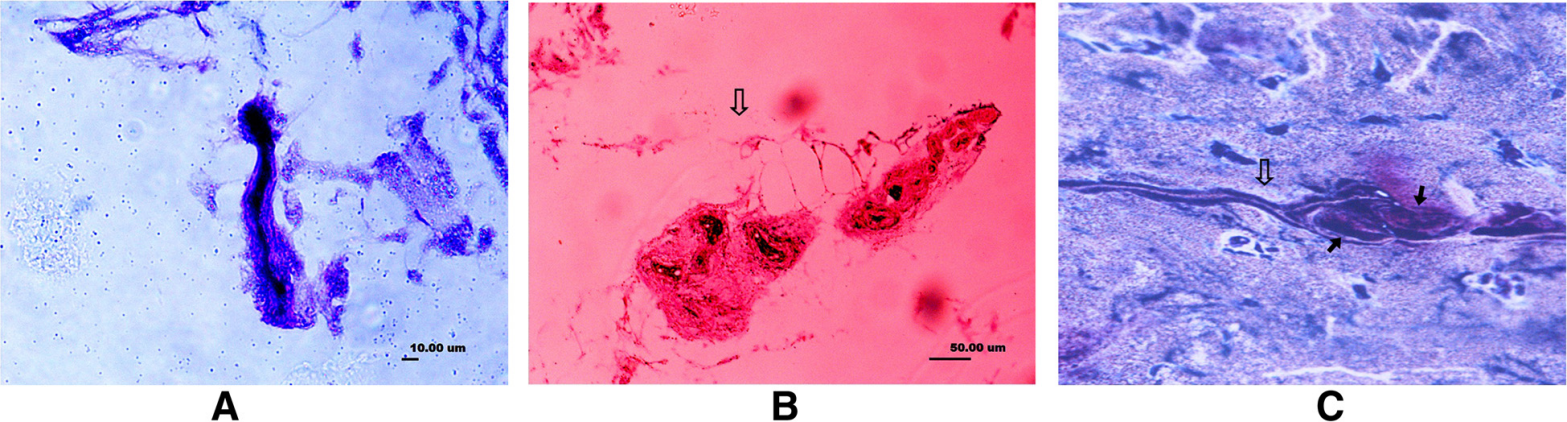
**a** A longitudinal section of a Pacini (type II). *Dark purple* indicates the cylindrical nerve fibers in the corpuscle, which is surrounded by layers of flat cells (×400). **b** A Pacini corpuscle formed in a botryoidal cluster, paralleling to long axis of ligaments (cross section). *Hollow arrow* indicates the dendritic nerve fibers (×400). **c** A blood vessel in nerve fiber of Pacini corpuscle (*hollow arrow*). Nerve fiber ends at purple-dyed oval-shaped corpuscle (*solid arrow*) (×400)

The Pacini were found to be thickly myelinated coniform or broom-shaped corpuscles. It was also found in single or as botryoidal cluster formed by more than ten corpuscles connected by axonal fibers (Fig. 4a), with the size of 150 × 40 μm. The dark purple-dyed cylindrical structure was the axonal fibers stretched into the Pacini, with the diameter of 5–10 μm. As more than ten concentric circles of darkly dyed flat cells were surrounded by an axonal fiber, the Pacini had another name of lamellar corpuscle. The morphological structure of an axonal fiber surrounded lamellar layers appeared to be diverse. Concordant to other reports, direction of Pacini axon had the same direction parallel to the direction of collateral ligaments (Fig. 4b). A large number of Pacini existed in the synovial tissues and the ligament at both ends of the collateral ligaments, which were accompanied by the nerve vessel bundles (Fig. 4c). Basically, the Pacini in the synovial and ligament tissue intervals were in smaller size than those in the ligament parenchyma. We found that the Pacinian corpuscles are the most common mechanoreceptors in all the ankle collateral ligaments.

**Fig. 5:**
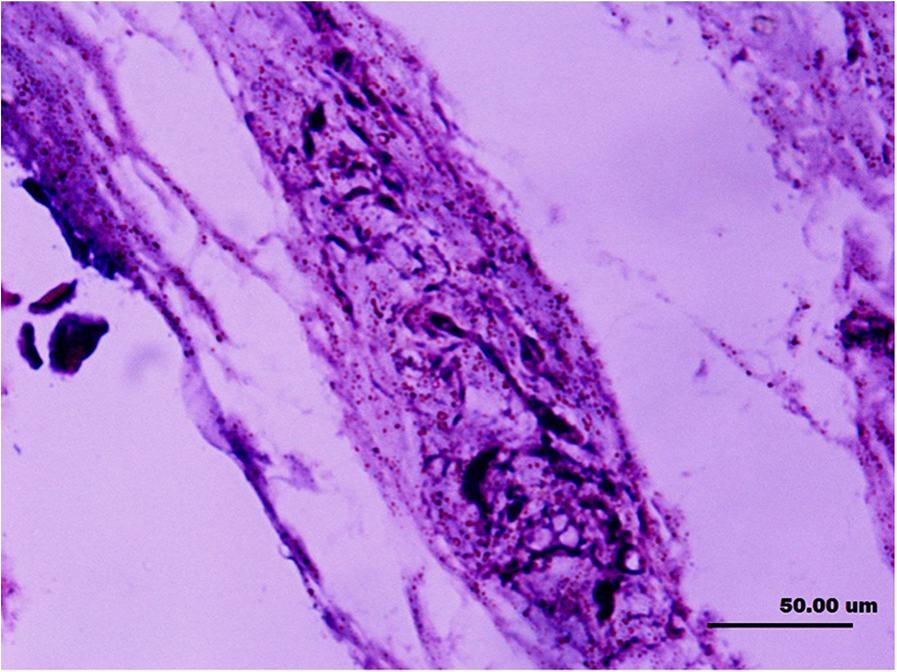
A longitudinal section of a Golgi tendon organ (type III), spindle-shaped with large volume. The *dark purple* mass indicates the winded nerve fibers (×400)

Golgi tendon organs (type III) were thinly myelinated spindle-shaped corpuscles (Fig. 5). It had the largest volume among the four types, with the mean size of 300 × 70 μm. The Golgi tendon organs were dyed bluish violet, with darker dyed shapelessly nerve substances. They existed singly or connected by nerve fibers. Although the Golgi tendon organs were found in all the ligaments, in this study, they were found less than Ruffini, which was not concordant with the literatures.

**Fig. 6:**
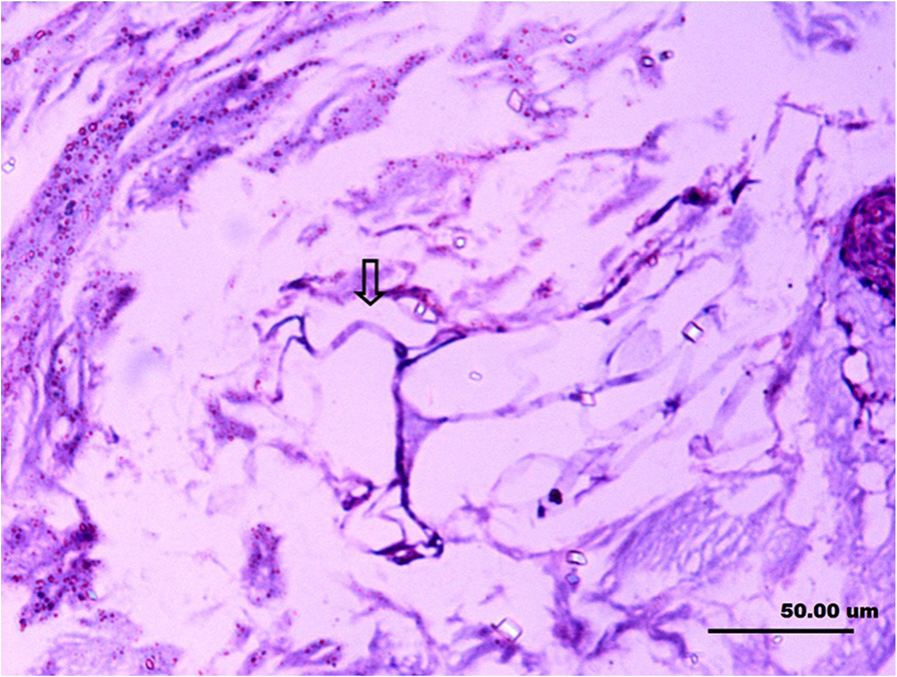
Free nerve endings (type IV). The *hollow arrow* indicates the nerve fiber (×400)

Free nerve endings (type IV) were non-myelinated fibers which were branched from an axonal fiber (Fig. 6). They had a diameter of 1–3 μm and were dyed lighter and lighter when got closer to the end. The amount of free nerve endings was found least in the collateral ligaments in this study.

### Statistical analysis of distribution of each type of receptor in the ligaments

#### Quantity statistics of mechanoreceptors of each type in the collateral ligaments of the ankle

**Fig. 7:**
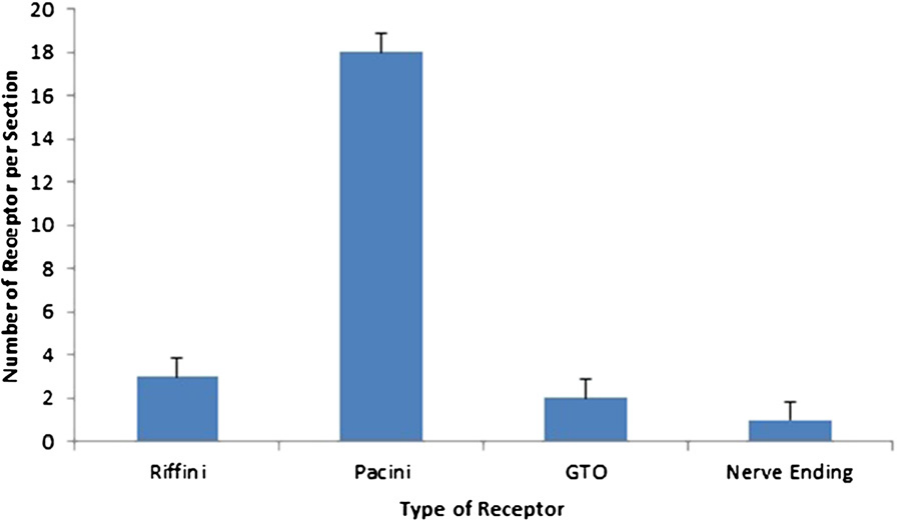
Comparison of sensory receptors in the ankle collateral ligament (mean ± SD)

**Fig. 8:**
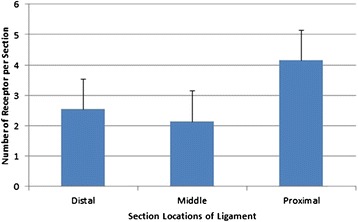
Segmental distributions of sensory receptors in the ankle collateral ligament (mean ± SD)

As is shown in Tables 1 and 2, type II (Pacini corpuscle) was predominant in the number of all four types of sensory receptors. The number of Pacini corpuscle was significantly higher than other receptors (one-way ANOVA, *p* < 0.01) (Figs. 7 and 8 and Tables 2 and 3).

**Table 2:** Number comparisons of mechanoreceptors of each type

Type	Number of sections	Mean ± SD
I	252	3 ± 0.86
II	252	18 ± 0.91
III	252	2 ± 0.89
IV	252	1 ± 0.81

**Table 3:** Comparisons of each type in the calcaneus fibula ligament

Type	Sections	Mean ± SD
I	108	5 ± 0.81
II	108	18 ± 0.88
III	108	2 ± 0.80
IV	108	1 ± 0.82

## Discussion

### Morphology and classification of mechanoreceptors in lateral ligaments of the human ankle

Mechanoreceptors have been identified in both human and animal joints [17–19]. In the cat knee, the mechanoreceptors were identified in joint capsule as well as in ligament parenchyma [17]. In human, the research of mechanoreceptors has been focused on large joints such as the knee and hip joint, only a few research focused on the collateral ligaments of ankles. Research on the morphological description and classification of mechanoreceptors in the ankle collateral ligaments have also been performed long time ago [20, 21]. New emerging technologies have produces different staining techniques, the mechanisms of classification, the morphological variation, and the integrity of neuroanatomy and electrophysiology. In this study, we used gold-chloride staining methods due to this method is a cost-effective methods, yet providing a clear view of mechanoreceptors in the ligament.

However, the injury of the lateral ligaments of the ankle is very common in clinic, taking up about 16 % of the sports injuries in emergency [22]. In the US, the incidence rate is one in ten thousand every day [23], 10 % of the patients developed instability of the ankle joint, repeated sprain, and even osteoarthritis after injury. Moreover, the previous research focused on the ankle anatomy and biomechanics, neglecting the proprioceptive function, due to the limitation of morphological and neurophysiological evidence. In recent years, the ankle disorders, especially the proprioceptive loss and restore in ankle instability, have attracted more research interests in orthopedics.

The staining technique is the key to success of the studies. For a long time, the immunohistochemistry and silver staining have been applied to the studies on morphology of mechanoreceptors. To avoid the high cost and relatively complicated procedures, we compared different staining methods in the literatures and chose modified O’Connor and Gonzales gold-chloride staining method. This staining method can clearly demonstrate the morphology of mechanoreceptors, suggestive of a better way of staining nerve endings.

Mechanoreceptors have diverse morphology, in addition to the typical four types. The classification of mechanoreceptors was according to Freeman and Wyke [17], which was widely accepted, as it precisely described the morphological features and functions of major mechanoreceptors in category. In our study, all types of mechanoreceptors in collateral ligaments were found one to three times smaller than described (Table 1). Moreover, the morphology and configuration of receptors in this study were different from typical four types reported in the literatures. For an instance, the Ruffini in human ankle ligaments are spindle shaped, differing from the widely accepted spherical shape. In addition, capillaries were found in some nerve fibers of Ruffini in this study, which suggested that Ruffini were closely related to blood circulation. Broom-shaped and botryoidal Pacini have not been reported before. Concordant with some reports [24], mechanoreceptors with transitional morphology and structure were found in this study. This may be caused by variation of classic types or possible new types. The diversity of morphology suggests the complication of proprioceptive function, which requires further study.

The different morphologies of mechanoreceptors may be caused by the different subjects, in addition to staining technique. Animal joint capsule receptor morphology may be different from the human. As mentioned before, Freeman and Wyke observed animals like cat in their research, mechanoreceptors were larger than human beings in their studies. This indicates that during evolution, the proprioceptive function of human beings may have degenerated. The ankle activities of human beings are more complicated and take more proportion of the body weight, while animals that walk with four limbs shared fewer loads on hind ankles. This may result in the diversity of mechanoreceptors in human ankle ligaments. However, more evidences and research are required to prove this assumption. Another reason for the different morphologies can be that we used collateral ligaments of the ankle, they differ from anterior or posterior cruciate ligament in other studies. Ligaments in different locations may lead to difference in mechanoreceptor morphology.

### The distribution and the proprioceptive function of mechanoreceptors

As a result from the controversy of the morphology and classification of mechanoreceptors, the amount and distribution of each type in the ligaments are disputed. It was reported that the types I and II mainly located in the joint capsule, rarely in ligaments. Michelson and Hutchins [19] failed to find type IV in human ankle ligaments, which was observed in cat ankle ligaments by different researchers [25]. Recently, Moraes identified a bunch of free nerve endings in lateral ligaments of the human ankle [18]. Some researchers failed to identify the Pacini in joints [24]. However, all typical four types of mechanoreceptors were observed in this study.

Concordant with most of the reports [17, 19, 25], the Pacini were found to have the largest amount and widest distribution in this study. They largely exist in the synovial tissue at both ends of ligaments and the tissue intervals branching into the ligaments, accompanied by nerve vessel bundles. The anatomic structures were assumed to form a tension-compression device [17]. The longitudinal tension of the ligament can compress the space of the fibrous septum, stimulating the mechanoreceptors. In this study, the long axis of the flat cells annularly wrapped Pacini was found parallel to the long axis of ligaments. This may provide an evidence for the tension-compression assumption. Although the Pacini morphology has been accepted by the majority of researchers, the distribution is still disputed. Some believed that the Pacini only existed in the loose connective tissues at both ends of the ligaments and the tissue intervals [17]. In this study, the Pacini were identified in the whole ligament parenchyma, in addition to the synovial tissues at the distal and proximal parts of ligaments.

Statistics showed that the amount of mechanoreceptors in the distal, middle, and proximal segments of the collateral ligament had no significant difference. However, if the mechanoreceptors in synovial membrane were counted in, the results would be different. The same situation happened to the type III, which existed more in synovial membrane than in the ligament parenchyma. As a result, the statistics showed less of type III than reported [18, 19], even less than type II.

In this study, we focused on the morphology, classification, and distribution of mechanoreceptors in collateral ligaments of the human ankle. The results could provide morphological evidence for the proprioceptive mechanoreceptors in the ligaments, which could help with the treatment of lateral ankle sprain and chronic ankle instability. Further research of the function of mechanoreceptors may lead to new strategies of treating ligament injuries of ankle joints.

## Conclusion

In this study, all the four typical types of mechanoreceptors were identified in the lateral ligament of the human ankle. However, the morphology and distribution were different from reported in the literatures, which indicated more complicated proprioceptive functions of mechanoreceptors in human beings.
